# The association between Lifelines Diet Score and GDM: a case-control study

**DOI:** 10.3389/fnut.2025.1625903

**Published:** 2025-07-30

**Authors:** Mohammad Othman Abudari, Nahlah Fahad Alreshidi, Saud Salman Alharbi, Shatha Hallal Alziyadi, Mansuor A. Alanazi, Nahla Kambal, Fatma Mohamed Elmansy, Mohamed Goda Elbqry

**Affiliations:** 1 Community Health Nursing Department, School of Nursing, The University of Jordan, Aqaba, Jordan; 2 Department of Internal Medicine, University of Haill, Hail, Saudi Arabia; 3 Department of Family and Community Medicine, Faculty of Medicine, University of Tabuk, Tabuk, Saudi Arabia; 4 Obstetrics and Gynecology Department, College of Medicine, University of Taif, Taif, Saudi Arabia; 5 Department of Clinical Nutrition, Jazan University, Jazan, Saudi Arabia; 6 Department of Medical and Surgical, College of Nursing, Qassim University, Buraidah, Saudi Arabia

**Keywords:** Lifelines Diet Score, LLDS, gestational diabetes mellitus, GDM, dietary physical activity, smoking, gravidity, family history of GDM

## Abstract

**Background:**

Gestational diabetes mellitus (GDM) is a common pregnancy-related complication with rising global prevalence, posing significant short-and long-term health risks to both mothers and their offspring. Various lifestyle and dietary factors have been implicated in its development. While dietary quality indices like the Lifelines Diet Score (LLDS) have shown promising associations with improved cardiometabolic outcomes, their relationship with GDM remains unclear. This study examines the relationship between the Lifelines Diet Score and the odds of gestational diabetes mellitus.

**Methods:**

This case-control investigation was conducted at a tertiary care referral center, specifically Qassim University Hospital, with participant enrollment occurring from January 2022 to January 2025. The study cohort consisted of 150 cases and 150 matched controls. Individual food consumption was documented to compute the LLDS utilizing a semiquantitative food frequency questionnaire.

**Results:**

A total of 300 participants (150 cases and 150 controls) were included. No significant differences were observed between groups regarding age, BMI, physical activity, smoking status, or education level. However, the case group had significantly higher gravidity (*p* = 0.024) and a greater family history of GDM (*p* = 0.041). Higher LLDS quartiles were associated with healthier nutrient profiles and favorable food group consumption, including increased intake of vegetables, fruits, legumes/nuts, and decreased intake of red/processed meats and sugar-sweetened beverages (P-trend < 0.05). Multivariable logistic regression showed a significant inverse association between LLDS and odds of GDM. Compared to the lowest quartile, participants in the highest LLDS quartile had 76% lower odds of GDM (adjusted OR: 0.23, 95% CI: 0.12–0.40; P-trend < 0.001).

**Discussion:**

Our findings indicate that greater adherence to the LLDS may be associated with reduced odds of GDM. Although the case-control design precludes causal inferences, these results highlight the potential significance of overall dietary quality in maternal metabolic health. Further prospective and intervention studies are warranted to validate these associations and elucidate the underlying mechanisms through which a high-quality diet may mitigate the odds of GDM.

## Introduction

Gestational diabetes mellitus (GDM) is a glucose intolerance that is first recognized during pregnancy and normally resolves after delivery (1). The condition affects up to 14% of all pregnancies worldwide, which varies across populations and diagnostic criteria (2). Globally, the prevalence of GDM is increasing, and this increases public health concerns associated with its short-and long-term implications for mothers as well as offspring.

Several factors, such as increasing maternal age and increasing rates of obesity, as well as sedentary lifestyles, have been attributed to the increasing prevalence of GDM (3). A recent study reported that the prevalence of gestational diabetes increased by 70% over 13 years, with more women requiring insulin treatment, but birth weights decreased (4).

In recent years, several studies have been conducted to investigate the relationship between the Lifelines Diet Score (LLDS) and several health outcomes. Women with higher adherence to LLDS have a lower likelihood of metabolically healthy obesity (5). For postmenopausal women, higher LLDS tertiles have been associated with reduced odds of hypertension and type 2 diabetes mellitus (6). It has been shown that greater adherence to LLDS is associated with lower systolic blood pressure and, in overweight and obese adults, a near-significant reduction in triglyceride levels, suggesting a protective effect against metabolic syndrome (7). These studies yield promising associations between LLDS and cardiometabolic health; however, research on its relation with other health conditions continues. For example, an ongoing study has looked at the relationship between LLDS and polycystic ovary syndrome (PCOS) in Iranian women (8). These findings further add support to the possible use of LLDS as a method to assess diet quality and its effect on a host of health outcomes.

Despite these findings, some studies have reported conflicting results regarding the relationship between dietary patterns and the odds of GDM (9). For example, even while some studies have shown a protective effect of healthy dietary patterns, others have not demonstrated significant correlations (10). The differences in these studies may stem from their differences in study design, characteristics of their populations, the dietary assessment methods used, and the confounder types they address. Therefore, further research is necessary to establish the connection between dietary patterns and the odds of GDM.

The LLDS is an innovative tool to evaluate the quality of the diet according to dietary guidelines (11). It includes different food groups and nutrients; hence, it can count as a total index of an individual’s dietary habits. Several health outcomes have been associated with the LLDS, such as cardiovascular diseases and type 2 diabetes mellitus (12). Despite this, the association with GDM has not been well characterized.

With the increasing prevalence of GDM and associated health risks, any modifiable risk, such as diet, must be identified to prevent it. The relationship between the LLDS and GDM odds may be understood and used to inform the development of dietary interventions to decrease GDM incidence.

Therefore, this study aims to examine the relationship between the Lifelines Diet Score and the odds of GDM in a case-control study design. We examine this relationship to make a contribution to the existing body of knowledge on dietary patterns and GDM in order to inform dietary recommendations for pregnant women aimed at reducing the odds of GDM.

## Method

### Study population

This case-control study was conducted at a tertiary care referral center, namely Qassim University Hospital, with participant enrollment occurring from January 2022 to January 2025. The study cohort consisted of 150 cases and 150 matched controls. Cases were defined as gravid women diagnosed with GDM according to standardized diagnostic protocols. Diagnosis was established via a 75-gram oral glucose tolerance test (OGTT), adhering to the International Association of Diabetes and Pregnancy Study Groups (IADPSG) thresholds: fasting plasma glucose ≥92 mg/dL, 1-h glucose ≥180 mg/dL, or 2-h glucose ≥153 mg/dL. Participants were systematically recruited during routine antenatal visits, primarily between 24 and 28 weeks of gestation—the clinically recommended period for GDM screening. To maintain cohort homogeneity, exclusion criteria were rigorously applied. Women with pre-existing diabetes mellitus (type 1 or type 2), multifetal gestations, or underlying metabolic disorders (e.g., polycystic ovary syndrome) were excluded. Additionally, individuals with a history of bariatric surgery or those on pharmacotherapies known to perturb glycemic regulation (e.g., corticosteroids) were ineligible. Consecutive enrollment was employed to mitigate selection bias and enhance sample representativeness (13). Participants who were following a special diet (such as therapeutic diets, vegetarian diets, weight-loss diets, or diabetes-specific diets), or participants who had modified their diet after being informed of their gestational diabetes diagnosis were excluded from the study.

Controls were drawn from the same obstetric population, consisting of normoglycemic pregnant women without GDM, confirmed by OGTT results below the diagnostic cutoffs. To minimize confounding, controls were matched to cases by maternal age (±5 years), gestational age (±2 weeks), and parity. Recruitment occurred concurrently in identical clinical settings to ensure temporal and environmental consistency. The exclusion criteria for the control group mirrored those established for the case group, thereby precluding individuals with pregestational diabetes, multifetal pregnancies, or metabolic comorbidities, including thyroid disorders (such as hypothyroidism or hyperthyroidism), chronic hypertension, chronic renal disease, and chronic hepatic disease. Additionally, women who conceived through assisted reproductive technology (ART) were excluded from the study to minimize potential confounding factors related to the metabolic and hormonal differences associated with ART pregnancies. Consecutive sampling was utilized to prevent selection bias and ensure demographic and clinical comparability. This matching strategy facilitated a robust examination of potential associations between exposures and GDM while controlling for key covariates.

The required sample size for this study was calculated based on previous research examining the association between dietary patterns and gestational diabetes (14, 15). Considering a significance level of 0.05, a statistical power of 80%, and the expected effect size, a minimum of 150 participants was estimated for each group (cases and controls). This sample size was deemed sufficient to detect meaningful differences in diet scores between the groups and to ensure the statistical validity of the study findings.

### Dietary assessment

Dietary intake was assessed using a semi-quantitative food frequency questionnaire (FFQ) comprising 152 food items, the validity of which has been previously established. In the validation study, the FFQ included 152 food items and showed acceptable validity, with correlation coefficients for nutrient intakes ranging from 0.2 to 0.7 compared to 24-h recalls. Cross-classification analysis indicated that over 70% of participants were classified into the same or adjacent quartiles by both methods, and Bland-Altman analysis showed no systematic bias. These results support the use of the FFQ as a valid tool for dietary assessment in this population (16). Participants reported their habitual consumption frequency for each item over the preceding 12-month period, selecting from predefined response categories spanning “never or less than once monthly” to “six or more times daily.” Nutrient intake quantification was performed using Nutritionist IV software, which enabled the computation of total energy intake and macronutrient/micronutrient composition based on standardized food composition databases (17). This approach facilitated the systematic evaluation of dietary patterns while accounting for variations in portion sizes and consumption frequencies.

### Lifelines Diet Score (LLDS)

The LLDS was calculated using the methodology described by Vinke et al. (18). In this scoring system, food groups were classified according to LLDS guidelines into categories with positive, negative, neutral, or unknown health effects, where neutral (e.g., eggs) and unknown food groups (including potatoes, refined grain products, white unprocessed meat, cheese, ready-to-eat savory products, sugary products, soups, sweetened dairy, and artificially sweetened products) were excluded from LLDS calculation. Nine food groups—vegetables, fruits, whole grain products, legumes and nuts, fish, oil and soft margarine, unsweetened dairy products, coffee, and tea—were classified as having positive health effects. Additionally, three food groups—red and processed meat, butter and hard margarine, and sugar-sweetened beverages—were categorized as having negative effects. To account for differences in energy intake between individuals and better reflect dietary quality, food consumption was expressed as grams per 1,000 kcal rather than grams per day for each food group. Individual intake levels were then divided into quintiles ranging from one (minimum consumption) to five (maximum consumption) for each food group. The total LLDS score, ranging from 12 to 60, was derived by summing the scores of all 12 components.

It is important to note that the classification of certain foods in the LLDS, such as margarine, potatoes, or coffee, may not fully align with cultural dietary patterns or metabolic responses in pregnant women from Middle Eastern populations. For instance, potatoes are a staple in many regional diets and may have different glycemic impacts depending on preparation methods and genetic factors influencing glucose metabolism. Similarly, coffee, while considered a positive component in LLDS, has complex metabolic implications in pregnancy. The LLDS, developed in Western populations, may thus require cultural adaptation and validation before full application in non-Western settings (18–20).

### Other assessment

A trained interviewer administered all questionnaires to ensure accurate and consistent responses from participants. Demographic and clinical data were collected using a structured Questionnaire, which included variables such as age (years), educational status, family history of GDM (yes/no), and smoking status (yes/no). Anthropometric measurements were obtained using standardized protocols: weight was measured to the nearest 0.1 kg using a calibrated digital Seca scale (Germany), with participants wearing lightweight clothing and no footwear; height was recorded barefoot in a standing position using a fixed stadiometer. Body mass index (BMI) was calculated as weight (kg) divided by height squared (m^2^). Waist circumference (WC) was measured at the narrowest point between the lower rib and iliac crest using a nonelastic tape measure, ensuring no compression of the skin. The combination of five variables, including education (academic = 1 and non-academic education = 0), family size (≤4 people = 1, > 4 people = 0), house acquisition (house ownership = 1, Lack of ownership = 0), foreign travel (yes = 1, no = 0), and income (high = 1, low and moderate = 0) were used to compute socioeconomic status (SES) score. Based on the frequency of SES scores in our study population, participants with SES score of 0 and 1, 2, 3–5 were classified as high, moderate, and low SES, respectively.

### Statistical analysis

Statistical analyses were performed using SPSS version 24.0 (IBM Corp., Armonk, NY), with the Shapiro-Wilk test used to assess data normality. For normally distributed variables, group comparisons were conducted using one-way ANOVA, while categorical variables were analyzed with Pearson’s chi-square test. For the comparison of means between the two independent groups (case and control), we used the independent samples t-test, as it is specifically designed to assess significant differences between two groups. For comparisons across LLDS quartiles (more than two groups), one-way analysis of variance (ANOVA) was applied. GDM odds was evaluated through binary logistic regression, calculating odds ratios (ORs) with 95% confidence intervals (95% CIs) after adjusting for confounders. Participants were categorized into LLDS score quartiles using rank ordering, which were then treated as categorical variables in regression models. Trend analysis was performed by assigning median values to each quartile as continuous variables. Results are presented as mean ± standard deviation, with statistical significance set at *p* < 0.05.

## Results

Table 1 presents the demographic characteristics of participants in the case and control groups. The mean age of participants was slightly higher in the case group (31.65 years) compared to the control group (30.87 years), though this difference was not statistically significant (*p* = 0.077). Similarly, the mean body mass index (BMI) was higher in the case group (29.35 kg/m^2^) than in the control group (27.81 kg/m^2^), but the difference did not reach statistical significance (*p* = 0.067). There was no significant difference in physical activity levels between the two groups (35.92 vs. 35.44 Met.h/week, *p* = 0.425). The proportion of smokers was also similar, with 7.33% in the case group and 4.66% in the control group (*p* = 0.754). However, a significant difference was observed in terms of gravidity (*p* = 0.024). A greater proportion of women in the control group were in their first pregnancy (G1: 63.4% vs. 36.6%), while higher gravidity (≥ G2) was more common in the case group (60.6% vs. 39.4%). Moreover, a significantly higher proportion of participants in the case group had a family history of gestational diabetes mellitus (GDM) compared to the control group (76.6% vs. 24.6%, *p* = 0.041). Educational status did not differ significantly between the two groups (*p* = 0.564), with the majority of participants in both groups reporting low or moderate education levels.

**Table 1:** Demographic characteristics of participants across case and control groups.

Continuous variables (mean ± SD)	Case (*n* = 150)	Control (*n* = 150)	*p*-value
Age (years)	31.65 ± 6.20	30.87 ± 5.82	0.077
Pre-pregnancy BMI (kg/m^2^)	27.41 ± 3.55	25.82 ± 3.12	0.053
BMI (kg/m^2^)	29.35 ± 4.19	27.81 ± 3.13	0.067
Physical activity (Met.h/week)	35.92 ± 8.85	35.44 ± 7.94	0.425

Categorical variables (*n*, %)	Case (*n* = 150)	Control (*n* = 150)	*p*-value
Smoking (yes)	11 (7.3%)	7 (4.7%)	0.754
Family history of GDM (yes)	115 (76.7%)	37 (24.7%)	0.041
Gravidity			0.024
G1	55 (36.7%)	91 (60.7%)	
≥ G2	95 (63.3%)	59 (39.3%)	
Socioeconomic status (SES)			<0.001
Low	32 (21.3%)	47 (31.3%)	
Middle	50 (33.3%)	60 (40.0%)	
High	68 (45.4%)	43 (28.7%)	
Education status			0.564
Illiterate	17 (11.3%)	14 (9.3%)	
Low education	73 (48.7%)	79 (52.7%)	
Higher education	60 (40.0%)	57 (38.0%)	

Continuous variables were compared using the independent samples *t*-test. Categorical variables were analyzed using the chi-square test.

Dietary assessment (Table 2) revealed distinct intake patterns between groups. GDM cases consumed significantly more energy (*p* < 0.05), total fat (*p* < 0.05), saturated fatty acids (*p* < 0.05), cholesterol (*p* < 0.05), carbohydrates (*p* < 0.05), sodium (*p* < 0.05), folate (p < 0.05), and iron (*p* < 0.05). Conversely, they had lower intake of monounsaturated (*p* < 0.05) and polyunsaturated fatty acids (*p* < 0.05), along with reduced consumption of potassium (*p* < 0.05), phosphorus (*p* < 0.05), calcium (*p* < 0.05), vitamin B12 (*p* < 0.05), and antioxidant micronutrients including zinc (*p* < 0.05), magnesium (*p* < 0.05), and vitamins E (*p* < 0.05), C (*p* < 0.05), and D (*p* < 0.05). Analysis by LLDS quartiles showed a significant positive association between higher LLDS scores and increased intake of protein (p-trend < 0.05), potassium (p-trend < 0.05), phosphorus (p-trend < 0.05), calcium (p-trend < 0.05), magnesium (p-trend < 0.05), zinc (p-trend < 0.05), and vitamins C (p-trend < 0.05) and D (p-trend < 0.05). Conversely, higher LLDS quartiles were inversely associated with total fat (p-trend < 0.05), MUFA (p-trend < 0.05), and PUFA consumption (p-trend < 0.05).

**Table 2:** Dietary intakes of participants across case-control groups as well as quartiles of LLDS.

Variables	Groups, mean (SD)		*p*-value^a^	Quartiles of LLDS, mean (SD)		*p*-value^b^
	Case (*n* = 150)	Control (n = 150)		Q1	Q4	
Energy (kcal/d)	2795.09 (805.02)	2494.64 (634.43)	<0.001	2701.06 (770.14)	2584.74 (669.86)	0.614
Carbohydrate (g/d)	378.18 (7.24)	348.18 (5.12)	0.001	358.19 (92.14)	366.58 (87.10)	0.724
Protein (g/d)	84.72 (1.51)	92.11 (1.58)	<0.001	81.97 (24.85)	95.15 (27.36)	<0.001
Fat (g/d)	111.88 (2.65)	94.46 (2.32)	<0.001	112.74 (49.42)	95.14 (29.78)	0.004
SFA (g/d)	35.56 (10.95)	31.84 (10.33)	<0.001	34.95 (14.21)	32.63 (10.44)	0.364
MUFA (g/d)	34.93 (13.29)	39.88 (15.97)	<0.001	35.89 (17.54)	27.56 (14.36)	<0.001
PUFA (g/d)	23.12 (11.35)	27.13 (14.29)	<0.001	28.85 (15.74)	21.59 (8.54)	<0.001
Cholesterol (mg/d)	296.16 (136.45)	264.52 (138.27)	0.009	278.62 (132.41)	289.51 (147.43)	0.904
Fiber (g/d)	40.60 (18.25)	42.53 (18.61)	0.311	42.83 (19.96)	41.40 (17.32)	0.371
Sodium (mg/d)	4743.38 (1826.95)	4309.7 (1884.62)	0.005	4850.45 (2145.35)	4267.81 (1467.76)	0.092
Potassium (mg/d)	3768.87 (1232.54)	4299.86 (1252.02)	<0.001	3487.15 (1187.41)	4728.26 (1191.41)	<0.001
Phosphor (mg/d)	1485.51 (488.65)	1620.12 (481.24)	0.006	1370.22 (414.22)	1748.46 (511.19)	<0.001
Iron (mg/d)	22.92 (8.96)	18.98 (6.24)	<0.001	21.02 (7.24)	21.14 (7.65)	0.825
Calcium (mg/d)	1218.43 (465.92)	1337.91 (459.76)	0.004	1086.94 (456.14)	1455.85 (497.25)	<0.001
Magnesium (mg/d)	372.7 (121.89)	405.55 (130.18)	0.005	338.48 (107.98)	445.36 (115.70)	<0.001
Zinc (mg/d)	14.4 (3.95)	15.59 (4.11)	0.002	14.13 (3.98)	16.16 (3.75)	<0.001
Vitamin C (mg/d)	161.8 (90.15)	200.51 (75.89)	<0.001	149.71 (80.51)	225.57 (82.78)	<0.001
Folate (mcg/d)	488.21 (165.28)	457.84 (160.17)	0.029	468.43 (181.66)	471.25 (135.20)	0.845
Vitamin B12 (mcg/d)	8.17 (3.47)	9.34 (4.43)	0.003	9.1 (5.86)	8.73 (2.54)	0.654
Vitamin E (mg/d)	20.28 (14.16)	26.23 (18.54)	<0.001	25.17 (16.11)	21.03 (10.84)	0.156
Vitamin D (mcg/d)	4.68 (2.44)	5.34 (2.06)	0.024	4.1 (1.81)	5.05 (2.17)	<0.001

SFA, saturated fatty acid. ^a^Obtained from t-test ^b^Obtained from ANOVA.

For the comparison across LLDS quartiles, only the first (Q1) and fourth (Q4) quartiles are presented in this table to highlight the differences between the lowest and highest adherence groups.

Table 3 presents the dietary intake of 12 LLDS components (measured in grams per 1,000 kcal) among participants stratified by case-control status and LLDS quartiles. Analysis revealed significant differences in dietary patterns between GDM patients and controls. Cases demonstrated significantly lower consumption of beneficial food groups, including vegetables (*p* < 0.05), fruits (*p* < 0.05), and legumes/nuts (*p* < 0.05), while showing higher coffee intake (*p* < 0.05). Among negative LLDS components, cases consumed significantly more red/processed meats (*p* < 0.05) and sugar-sweetened beverages (*p* < 0.05). When examining trends across LLDS quartiles, all positive components (with the exception of coffee and tea) showed significant increases (p-trend<0.05), while negative components exhibited significant decreases (p-trend<0.05).

**Table 3:** Dietary consumption of the 12 components included in the LLDS in grams per 1,000 kcal among case-control participants and quartiles of LLDS.

Variable	Groups, mean (SD)		*p*-value^a^	Quartiles of LLDS, mean (SD)		*p*-value^b^
	Case (*n* = 150)	Control (*n* = 150)		Q1	Q4	
LLDS score	36.98 (6.11)	39.68 (5.58)	<0.001	30.92 (2.45)	46.13 (3.04)	<0.001
Positive components						
Vegetables	115.86 (57.96)	131.12 (51.22)	0.002	91.73 (39.95)	162.17 (53.69)	<0.001
Fruits	170.69 (87.96)	189.70 (69.97)	0.004	142.96 (78.55)	227.26 (80.00)	<0.001
Whole grain products	38.33 (31.99)	35.58 (32.06)	0.314	26.30 (23.86)	44.08 (28.38)	<0.001
Legumes and nuts	14.81 (12.46)	18.89 (11.92)	<0.001	13.85 (12.56)	21.41 (12.11)	<0.001
Fish	6.76 (7.33)	7.65 (7.78)	0.061	5.51 (6.12)	9.36 (9.31)	<0.001
Oils and soft margarines	2.56 (2.98)	2.64 (2.99)	0.187	2.30 (2.37)	3.02 (3.31)	<0.001
Unsweetened dairy	196.44 (108.85)	199.85 (118.85)	0.641	150.12 (90.70)	244.47 (119.32)	<0.001
Coffee	0.03 (0.07)	0.00 (0.02)	0.006	0.00 (0.02)	0.02 (0.06)	0.301
Tea	1.49 (0.20)	1.47 (1.05)	0.589	1.42 (0.86)	1.52 (0.95)	0.847
Negative components						
Red and processed meat	15.24 (11.12)	13.27 (8.75)	0.002	18.17 (12.26)	12.07 (8.45)	<0.001
Butter, hard margarines	16.52 (11.68)	16.18 (11.02)	0.701	20.85 (11.76)	11.70 (7.99)	<0.001
Sugar-sweetened beverages	35.50 (39.34)	24.89 (25.83)	<0.001	47.81 (45.59)	18.44 (13.89)	<0.001

^a^Obtained from t-test. ^b^Obtained from ANOVA.

For the comparison across LLDS quartiles, only the first (Q1) and fourth (Q4) quartiles are presented in this table to highlight the differences between the lowest and highest adherence groups.

Table 4 presents the odds ratios (ORs) and 95% confidence intervals (CIs) for gestational diabetes mellitus (GDM) across quartiles of the food-based Lifelines Diet Score (LLDS). In the crude model, higher adherence to the LLDS was significantly associated with a lower odds of GDM. Compared to the lowest quartile (Q1), the ORs for GDM were 0.58 (95% CI: 0.33–0.93) in Q2, 0.49 (95% CI: 0.28–0.82) in Q3, and 0.34 (95% CI: 0.18–0.58) in Q4 (P for trend < 0.001). After adjusting for age and BMI in Model 1, the inverse association remained significant, with ORs of 0.50 (95% CI: 0.29–0.89) in Q2, 0.46 (95% CI: 0.26–0.77) in Q3, and 0.31 (95% CI: 0.16–0.54) in Q4 (P for trend < 0.001). Further adjustment for additional confounders, including energy intake, physical activity, smoking, gravidity, family history of GDM, SES, pre-pregnancy BMI and education status in Model 2, also showed a significant decreasing trend in GDM odds: ORs were 0.38 (95% CI: 0.22–0.80), 0.42 (95% CI: 0.23–0.74), and 0.23 (95% CI: 0.12–0.40) in Q2, Q3, and Q4, respectively (P for trend < 0.001).

**Table 4:** Odds ratio (OR) and 95% confidence interval (CI) for GDM based on quartiles of food-based LLDS.

Variable	Quartiles of LLDS**				*p* for trend
	Q1	Q2	Q3	Q4	
Crude model	1.00 (ref)	0.58 (0.33–0.93)	0.49 (0.28–0.82)	0.34 (0.18–0.58)	<0.001
Model 1*	1.00 (ref)	0.50 (0.29–0.89)	0.46 (0.26–0.77)	0.31 (0.16–0.54)	<0.001
Model 2†	1.00 (ref)	0.38 (0.22–0.80)	0.42 (0.23–0.74)	0.23 (0.12–0.40)	<0.001

**Binary logistic regression was used to obtain OR and 95% CI.

*Model 1: adjusted for age and BMI.

†Model 2: adjusted for model 1 and Energy, Physical activity, Smoking, Gravidity, Family history of GDM, SES, pre-pregnancy BMI and Education status.

## Discussion

This case-control study aimed to investigate the association between adherence to the LLDS and the odds of GDM. While our findings suggest that better adherence to the LLDS may be associated with a lower odds of developing GDM, it is important to interpret these results cautiously, given the observational nature of the study. Nonetheless, the results are aligned with the existing literature, which underscores the role of high-quality dietary patterns in mitigating adverse metabolic outcomes during pregnancy. These findings contribute to the expanding body of evidence advocating for the utility of LLDS as a comprehensive dietary quality index in metabolic health assessment (21).

Previous research has extensively explored the relationship between adherence to the LLDS and various chronic diseases. Khani-Juyabad et al. (22) demonstrated that higher LLDS adherence was significantly associated with a lower odds of cardiovascular disease mortality in the Dutch population. Chen et al. (23) also observed an inverse relationship between type 2 diabetes and amyotrophic lateral sclerosis; type 2 diabetes may have a neuroprotective effect. Shi et al. (11) found that higher LLDS scores were linked to decreased odds of lung cancer incidence and mortality, emphasizing its relevance in cancer prevention. Sohouli et al. (24) showed that greater adherence to LLDS was associated with reduced odds of breast cancer in a case-control study. Similarly, Cai et al. (25) identified an inverse association between LLDS adherence and the odds of chronic kidney disease in their prospective cohort study. Asiaei et al. (26) highlighted the role of LLDS in improving metabolic syndrome components, with higher adherence associated with favorable metabolic health profiles. Darabi et al. (27) demonstrated that greater LLDS adherence correlated with lower depression symptoms and improved quality of life among adolescents. Furthermore, Wang et al. (28) reported that adherence to a higher-quality diet, as indicated by the LLDS, was associated with a lower odds of developing hypertension in a cohort study.

These findings collectively suggest that LLDS is a robust and reliable indicator of overall metabolic and chronic disease risk across diverse populations and health outcomes. However, to the best of our knowledge, no prior study has specifically investigated the association between LLDS adherence and the odds of GDM, particularly within a Middle Eastern population. This underscores the novelty and significance of the present study.

The observed inverse association between LLDS adherence and odds of GDM may be explained by the specific consumption patterns characteristic of higher LLDS scores. Participants in the highest quartiles demonstrated increased intake of fruits, vegetables, whole grains, legumes, and nuts—foods rich in fiber, antioxidants, and phytochemicals that can improve glycemic control, reduce systemic inflammation, and modulate gut microbiota composition (29, 30). For example, dietary fiber slows glucose absorption and promotes satiety, factors critical to GDM prevention (31). Additionally, greater consumption of unsweetened dairy products, fish, and healthy fats, such as those from oils and soft margarines, was observed among participants with higher LLDS, contributing further to improved insulin sensitivity and metabolic outcomes (18).

Conversely, women with lower LLDS scores reported higher intake of red and processed meats, sugar-sweetened beverages, and saturated fats—dietary components known to exacerbate systemic inflammation and insulin resistance (32, 33). Processed meats and sugary drinks have also been linked to excessive gestational weight gain and dyslipidemia, reinforcing their role as modifiable factors for GDM (34).

An interesting observation was the higher coffee consumption among GDM cases compared to controls, despite coffee being considered a positive component in LLDS. While moderate coffee intake has been associated with reduced odds of type 2 diabetes in non-pregnant populations, its impact during pregnancy remains uncertain. Caffeine can cross the placental barrier and may influence fetal growth and glucose metabolism (19, 35). Genetic polymorphisms affecting caffeine metabolism may further modulate its effects (20), highlighting the complexity of dietary component classification during pregnancy.

Physiologically, coffee contains caffeine and various polyphenols that can influence glucose metabolism through multiple pathways. Caffeine has been shown to acutely increase catecholamine release and transiently impair insulin sensitivity by antagonizing adenosine receptors, which may lead to higher postprandial glucose levels in sensitive individuals (36, 37). Moreover, during pregnancy, the metabolism of caffeine slows significantly due to reduced activity of cytochrome P450 enzymes, particularly CYP1A2, resulting in prolonged fetal exposure. This may contribute to alterations in fetal glucose-insulin homeostasis (38). Additionally, some studies suggest that genetic polymorphisms (e.g., in the CYP1A2 or ADORA2A genes) modulate individual responses to caffeine, further complicating its classification as a uniformly “positive” dietary component in pregnancy (39, 40). Thus, while coffee is generally considered a healthful beverage in LLDS, its effects in pregnancy—especially among genetically susceptible or metabolically vulnerable individuals—warrant more nuanced evaluation.

Moreover, higher LLDS adherence was associated with greater intake of micronutrients such as magnesium, potassium, calcium, zinc, and vitamins C, D, and E—nutrients with known antioxidant and anti-inflammatory properties. Deficiencies in magnesium and vitamin D, in particular, have been implicated in impaired glucose metabolism and increased odds of GDM [25–28]. These associations further underline the relevance of diet quality, beyond macronutrient distribution, in maternal metabolic health.

Although the case group had higher intakes of carbohydrates, sodium, and iron compared to the control group, no significant differences were observed for these nutrients across the quartiles of the Lifelines Diet Score (LLDS). This apparent inconsistency may be due to the composite nature of the LLDS, which reflects overall dietary quality based on a range of food groups rather than focusing on individual nutrient intakes. As a result, individuals with similar LLDS may still have different patterns of specific nutrient consumption. Additionally, cultural dietary habits and local food preferences may influence the intake of certain nutrients independently of LLDS classification. Measurement errors or recall bias inherent to the use of food frequency questionnaires (FFQs) may also contribute to these findings. These factors should be considered when interpreting the lack of significant differences in some nutrient intakes across LLDS quartiles despite the observed differences between cases and controls.

Our findings are consistent with those of previous studies that used alternative diet quality indices, such as the Healthy Eating Index (HEI) and the Alternate Mediterranean Diet (aMED), which have similarly reported inverse associations with odds of GDM (12, 41).

Similar findings have been reported in diverse populations, which may support the broader applicability of our results. For instance, Bao et al. (42) found that higher adherence to a healthy dietary pattern, such as the Alternate Healthy Eating Index (AHEI), was associated with lower odds of GDM in a U. S. cohort of over 13,000 women. Similarly, Shin et al. (43) demonstrated an inverse association between a diet rich in fruits and vegetables and the odds of GDM in Korean women. A study by Karamanos et al. (44) across 10 Mediterranean countries also showed that adherence to a Mediterranean diet was significantly associated with lower GDM incidence. These comparisons strengthen the generalizability of our findings and highlight the relevance of diet quality across different cultural and genetic backgrounds. However, cultural adaptations of scoring systems like the LLDS may still be necessary to reflect regional dietary patterns.

A notable strength of this study is its focus on the LLDS in relation to GDM, representing one of the first investigations to explore this association in a Middle Eastern population. The use of a validated and culturally adapted FFQ and comprehensive adjustment for multiple potential confounders enhance the reliability of our findings. Additionally, the consideration of a wide range of nutrient intakes provides a deeper understanding of dietary patterns beyond simple macronutrient analysis. However, several limitations warrant consideration. A notable limitation of this study is the use of a retrospective Food Frequency Questionnaire (FFQ), which may be subject to recall bias and social desirability bias. Participants may not accurately remember or may intentionally misreport their dietary intake, especially over extended periods, leading to potential misclassification of food consumption. These biases could affect the validity and reliability of the dietary data collected and may influence the observed associations in our findings. Future studies employing more objective or prospective dietary assessment methods are recommended to minimize these limitations. Residual confounding by factors such as physical activity, sleep quality, stress, supplement use, gestational weight gain, and genetic predisposition cannot be fully excluded. The single-center recruitment limits generalizability, and potential cultural misclassification of certain LLDS components, such as potatoes and white rice, may influence results. A further limitation of this study is that we did not formally assess multicollinearity among the independent variables in the logistic regression models. This may have affected the stability and interpretability of the estimated associations. Future studies should consider evaluating multicollinearity to strengthen the robustness of the findings.

Although our findings support an inverse association between higher LLDS adherence and odds of GDM, it is critical to recognize the inherent limitations of causal inference in case-control studies. The retrospective design does not allow for temporality assessment, and therefore, causality cannot be established. Observed associations may be subject to residual confounding despite multivariable adjustments. Prospective cohort studies and randomized controlled trials are needed to confirm the directionality and causality of the observed relationship between dietary quality and odds of GDM.

One important limitation of this study is the potential for temporal bias and reverse causation, as dietary data were collected after the diagnosis of GDM. Although we attempted to minimize this bias by including only newly diagnosed GDM cases and excluding participants who had already adopted special diets prior to data collection, some risk of recall or temporal bias may still remain. Additionally, the retrospective assessment of dietary intake using a food frequency questionnaire (FFQ) may be subject to inaccuracies in participants’ recall. Therefore, our findings should be interpreted as associations rather than causal relationships, and further prospective studies are needed to confirm these results.

Data on gestational weight gain were not comprehensively collected, which limited our ability to control for this important risk factor for GDM. Additionally, family history of diabetes was recorded in general terms without distinguishing between type 1, type 2, or gestational diabetes, or specifying maternal versus paternal lineage, which may have reduced the precision of our adjustment for genetic risk.

## Conclusion

Our findings indicate that greater adherence to LLDS may be linked to reduced odds of GDM. Although the case-control design limits causal interpretations, these results underscore the potential importance of overall diet quality in maternal metabolic health. Given its simplicity and comprehensiveness, the LLDS could serve as a practical tool for early dietary evaluation and intervention in pregnancy. Further prospective and intervention studies are warranted to validate these associations and clarify the mechanisms through which a high-quality diet may reduce the odds of GDM.

## Data Availability

The raw data supporting the conclusions of this article will be made available by the authors, without undue reservation.
